# RIPK2 as a New Therapeutic Target in Inflammatory Bowel Diseases

**DOI:** 10.3389/fphar.2021.650403

**Published:** 2021-04-14

**Authors:** Hajime Honjo, Tomohiro Watanabe, Ken Kamata, Kosuke Minaga, Masatoshi Kudo

**Affiliations:** Department of Gastroenterology and Hepatology, Kindai University Faculty of Medicine, Osaka-Sayama, Japan

**Keywords:** inflammatory bowel diseases, pro-inflammatory cytokines, toll-like receptors, nuclear factor-kappa B, RIPK2

## Abstract

Inflammatory bowel diseases (IBDs) are becoming more frequent worldwide. A significant fraction of patients with IBD are refractory to various types of therapeutic biologics and small molecules. Therefore, identification of novel therapeutic targets in IBD is required. Receptor-interacting serine/threonine kinase 2 (RIPK2), also known as receptor-interacting protein 2 (RIP2), is a downstream signaling molecule for nucleotide-binding oligomerization domain 1 (NOD1), NOD2, and Toll-like receptors (TLRs). RIPK2 is expressed in antigen-presenting cells, such as dendritic cells and macrophages. Recognition of microbe-associated molecular patterns by NOD1, NOD2, and TLRs leads to the interaction between RIPK2 and these innate immune receptors, followed by the release of pro-inflammatory cytokines such as TNF-α, IL-6, and IL-12/23p40 through the activation of nuclear factor kappa B and mitogen-activated protein kinases. Thus, activation of RIPK2 plays a critical role in host defense against microbial infections. Recent experimental and clinical studies have provided evidence that activation of RIPK2 is involved in the development of autoimmune diseases, especially IBDs. In addition, the colonic mucosa of patients with IBD exhibits enhanced expression of RIPK2 and associated signaling molecules. Furthermore, the blockage of RIPK2 activation ameliorates the development of experimental murine colitis. Thus, activation of RIPK2 underlies IBD immunopathogenesis. In this review, we attempt to clarify the roles played by RIPK2 in the development of IBD by focusing on its associated signaling pathways. We also discuss the possibility of using RIPK2 as a new therapeutic target in IBD.

## Introduction

Inflammatory bowel diseases (IBDs) include Crohn’s disease (CD) and ulcerative colitis (UC), chronic inflammatory and relapsing disorders of the gastrointestinal tract that are becoming more frequent worldwide (Baumgart and Sandborn, 2012; Ungaro et al., 2017). Genetic susceptibility and intestinal dysbiosis together mediate dysregulated immune responses against the intestinal microbiota, in patients with IBD (Strober et al., 2007; Strober and Fuss, 2011; Caruso et al., 2020). It is now generally accepted that excessive release of pro-inflammatory cytokines such as TNF-α, IL-12/23p40, and IL-6 underlies the immunopathogenesis of IBDs (Strober et al., 2007; Strober and Fuss, 2011; Caruso et al., 2020). This notion has been fully supported by the fact that biologics and small molecules targeting pro-inflammatory cytokine signaling pathways have been used with remarkable success in the treatment of IBD patients (Neurath, 2014; Verstockt et al., 2018; Shivaji et al., 2020). However, a significant fraction of patients with IBD are refractory to various types of biologics and small molecules. Therefore, identification of novel therapeutic targets in IBD is required.

Genome-wide association studies have identified susceptibility loci for the development of CD. The loss-of-function mutations in the caspase activation and recruitment domain 15 (*CARD15*) gene encoding nucleotide-binding oligomerization domain 2 (NOD2) and in the autophagy related 16 like 1 (*ATG16L1*) gene are the strongest genetic risk factors for CD (Inohara et al., 2005; Cho, 2008; Chen et al., 2009; Strober and Watanabe, 2011; Jostins et al., 2012; Philpott et al., 2014). NOD2 is an intracellular receptor for muramyl dipeptide (MDP), a small molecule derived from gut bacterial wall components (Inohara et al., 2005; Cho, 2008; Chen et al., 2009; Strober and Watanabe, 2011; Philpott et al., 2014). On the other hand, ATG16L1 functions as a critical molecule for autophagy, which is an indispensable process for the digestion and degradation of invading gut pathogens (Mizushima et al., 2008; Virgin and Levine, 2009; Salem et al., 2015). Receptor-interacting serine/threonine kinase 2 (RIPK2), also known as receptor-interacting protein 2 (RIP2), is a signaling molecule downstream of NOD2 and ATG16L1 (Inohara et al., 2005; Chen et al., 2009; Cooney et al., 2010; Strober and Watanabe, 2011; Philpott et al., 2014; Honjo et al., 2021). Recent studies have provided evidence that excessive activation of RIPK2 is involved in the development of experimental and human IBD (Jun et al., 2013; Hofmann et al., 2020). Moreover, small molecules inhibiting the activation of RIPK2 both *in vitro* and *in vivo* have been identified. In this review article, we summarize RIPK2-mediated signaling pathways with a focus on pro-inflammatory cytokine responses and discuss the colitogenic roles played by RIPK2.

## Expression and Structure of Receptor-Interacting Serine/Threonine Kinase 2

RIPK2 is expressed in the cytoplasm of antigen-presenting cells (APCs), such as dendritic cells (DCs) and macrophages (Chen et al., 2009; Strober and Watanabe, 2011; Philpott et al., 2014; Strober et al., 2014), and is also expressed in T cells (Shimada et al., 2018). In addition to hematopoietic cells, epithelial cells also express functional RIPK2 (Inohara et al., 2005; Kobayashi et al., 2005; Chen et al., 2009; Philpott et al., 2014). Thus, innate immune cells, including APCs, and epithelial cells express functional RIPK2.

**FIGURE 1:**
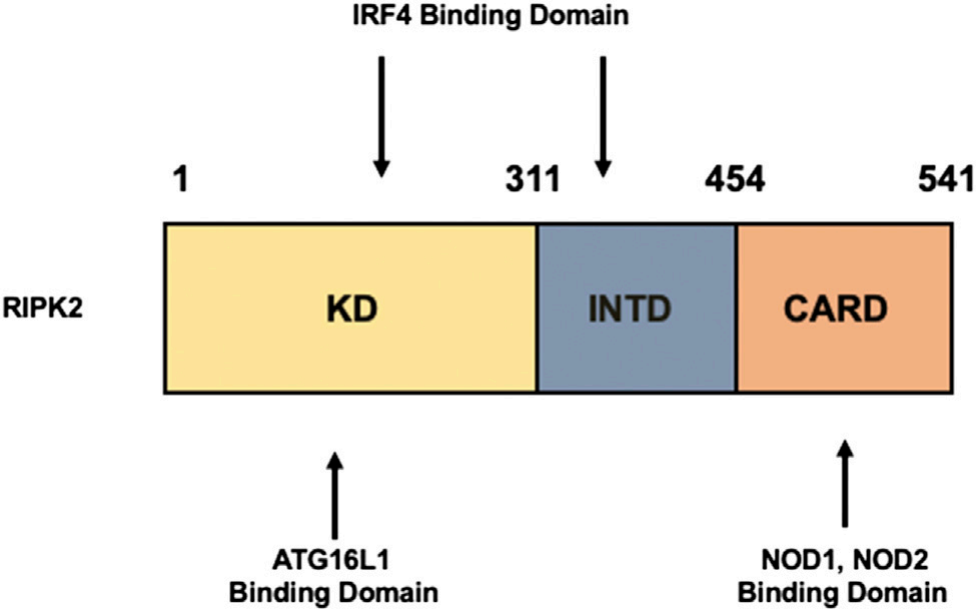
Structure of RIPK2. Receptor-interacting serine/threonine kinase 2 (RIPK2) is composed of a kinase domain (KD), an intermediate domain (INTD), and a caspase activation and recruitment domain (CARD). Interferon regulatory factor 4 (IRF4) binds to the KD and INTD. Autophagy related 16 like 1 (ATG16L1) binds to the KD, whereas nucleotide-binding oligomerization domain 1 (NOD1) and NOD2 interact with RIPK2 via CARDs.

RIPK2 is an obligate signaling molecule downstream of NOD1 and NOD2 (Chen et al., 2009; Strober and Watanabe, 2011; Philpott et al., 2014; Strober et al., 2014) (Park et al., 2007; Hall et al., 2008). It is composed of a kinase domain (KD), an intermediate domain (INTD), and a caspase activation and recruitment domain (CARD) (Figure 1). NOD1 and NOD2 bind to the CARD of RIPK2 through a CARD–CARD interaction (Strober et al., 2006; Chen et al., 2009; Strober and Watanabe, 2011; Philpott et al., 2014; Strober et al., 2014). A serine residue at position 176 in the KD is necessary for the activation of RIPK2 because a S176A RIPK2 mutant exhibited defective autophosphorylation and catalytic activity (Dorsch et al., 2006). Further, the interaction between ATG16L1 and RIPK2 is mediated by the KD (Honjo et al., 2021) whereas interferon regulatory factor 4 (IRF4) binds to the KD and INTD of RIPK2 (Watanabe et al., 2008; Watanabe et al., 2014). Thus, each domain of RIPK2 plays an indispensable role in protein–protein interactions.

## Activation of Receptor-Interacting Serine/Threonine Kinase 2 by Pattern Recognition Receptors

**FIGURE 2:**
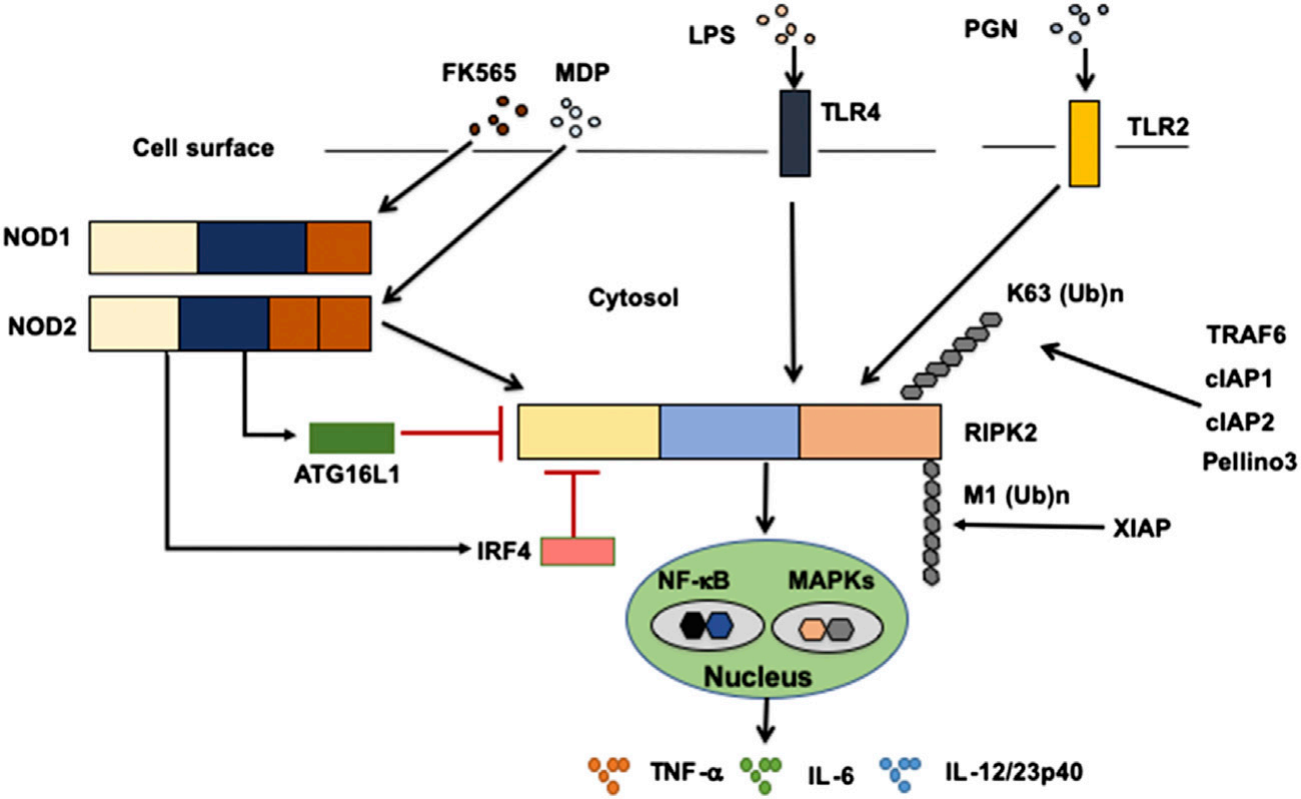
Signaling pathways mediated by RIPK2. Receptor-interacting serine/threonine kinase 2 (RIPK2) is a signaling molecule downstream of nucleotide-binding oligomerization domain 1 (NOD1), NOD2, and Toll-like receptors (TLRs). Activation of RIPK2 by TLRs results in a robust production of pro-inflammatory cytokines, including TNF-α, IL-6, and IL-12/23p40, through the nuclear translocation of nuclear factor-κB (NF-κB) subunits. Cellular inhibitor of apoptosis protein 1 (cIAP1), cIAP2, TNF receptor-associated factor 6 (TRAF6), Pellino 3 and X-linked inhibitor of apoptosis protein (XIAP) ubiquitinate RIPK2. RIPK2 is subjected to lysine 63 (K63)-linked polyubiquitination by TRAF6, cIAP1, cIAP2, and Pellino3. RIPK2 is also subjected to N-terminal methionine (M1)-linked polyubiquitination by XIAP. Interferon regulatory factor 4 (IRF4) and autophagy related 16 like 1 (ATG16L1) negatively regulate RIPK2-mediated pro-inflammatory cytokine responses. LPS, lipopolysaccharide; PGN, peptidoglycan, MDP, muramyl dipeptide.

Sensing of microbe-associated molecular patterns (MAMPs) activates cell surface TLRs as well as cytosolic NOD1 and NOD2 (Takeda and Akira, 2005; Strober et al., 2006; Strober and Fuss, 2011). Recognition of TLR and NOD1/NOD2 ligands by these pattern recognition receptors leads to a subsequent activation and ubiquitination of RIPK2 followed by the nuclear translocation of nuclear factor κB (NF-κB) subunits and activation of mitogen-activated protein kinases (Takeda and Akira, 2005; Strober et al., 2006; Strober et al., 2007; Strober and Fuss, 2011) (Figure 2). TGF-β–activated kinase 1 (TAK1) is a critical downstream signaling molecule for RIPK2-mediated production of pro-inflammatory cytokines such as IL-6, TNF-α, and IL-12/23p40 (Takeda and Akira, 2005; Strober et al., 2006; Strober et al., 2007; Strober and Fuss, 2011). Signaling pathways mediated by RIPK2 have been well studied in terms of NOD1 and NOD2 activation. Macrophages isolated from RIPK2-deficient mice exhibited defective pro-inflammatory cytokine responses upon stimulation with NOD1 and NOD2 ligands, but not with TLR ligands (Park et al., 2007; Hall et al., 2008), suggesting that cytokine responses mediated by NOD1 and NOD2 are dependent on RIPK2. Therefore, it is established that RIPK2 functions as a critical signaling molecule in the production of pro-inflammatory cytokines mediated by NOD1 and NOD2.

However, others have demonstrated RIPK2-dependent TLRs-induced pro-inflammatory cytokine production (Kobayashi et al., 2002; Lu et al., 2005; Dorsch et al., 2006; Usluoglu et al., 2007). In fact, the molecular interaction between TLRs and RIPK2 was observed after the stimulation with TLR ligands (Kobayashi et al., 2002; Lu et al., 2005). Moreover, macrophages isolated from RIPK2-deficient mice exhibited markedly reduced production of IL-6 and TNF-α upon the stimulation with TLR2 and TLR4 ligands compared with that observed in stimulated macrophages from RIPK2-intact mice (Kobayashi et al., 2002). Another report showed that activation of RIPK2 was involved in the production of IL-12/23p40 in human DCs stimulated with TLR4 ligands (Usluoglu et al., 2007). Therefore, these studies suggest that RIPK2 functions as a critical signaling molecule for the production of IL-6, TNF-α, and IL-12/23p40 mediated by TLRs and NOD1/NOD2. It should be emphasized that the magnitude of pro-inflammatory cytokine responses mediated by the TLRs–RIPK2 pathway is much greater than that of the responses mediated by the NOD2–RIPK2 pathway (Watanabe et al., 2004). Such strong pro-inflammatory responses mediated by the TLRs–RIPK2 pathway are negatively regulated by IRF4 and ATG16L1 (see below). Involvement of RIPK2 in TLRs-mediated signaling requires evaluation in future studies, because the current reports fail to clarify the role of RIPK2 in TLRs-mediated signaling pathways (Park et al., 2007; Hall et al., 2008).

In addition to the activation of NF-κB, RIPK2 is involved in type I IFN signaling pathways. Sensing of MAMPs by NOD1 leads to robust production of IFN-β by gastric epithelial cells in a RIPK2-dependent manner (Watanabe et al., 2010). Upon its activation, RIPK2 binds to TNF receptor-associated factor 3 (TRAF3), followed by the activation of TANK-binding kinase 1 and IκB kinase ε, as well as the subsequent nuclear translocation of IRF7. These processes subsequently trigger robust production of IFN-β. Furthermore, type I IFN responses mediated by the NOD1–RIPK2–TRAF3–IRF7 axis play critical roles in mucosal host defense against *Helicobacter pylori* infection of the stomach (Watanabe et al., 2010).

## Ubiquitination of Receptor-Interacting Serine/Threonine Kinase 2

Activation of RIPK2 is tightly regulated by polyubiquitination (Yang et al., 2007; Hasegawa et al., 2008; Bertrand et al., 2009; Tao et al., 2009; Bertrand et al., 2011; Damgaard et al., 2012; Damgaard et al., 2013; Yang et al., 2013; Hrdinka et al., 2016), a post-translational modification that determines the activation or degradation of a target protein (Wullaert et al., 2006). It is well established that lysine 48 (K48)- and K63-linked polyubiquitination reactions induce degradation and activation of ubiquitinated proteins, respectively (Wullaert et al., 2006). RIPK2-mediated activation of NF-κB depends on RIPK2 K63–linked polyubiquitination by various E3 ligases, including cellular inhibitor of apoptosis protein 1 (cIAP1), cIAP2, X-linked inhibitor of apoptosis protein (XIAP), Pellino 3, and TRAF6 (Yang et al., 2007; Hasegawa et al., 2008; Bertrand et al., 2009; Tao et al., 2009; Bertrand et al., 2011; Damgaard et al., 2012; Damgaard et al., 2013; Yang et al., 2013; Hrdinka et al., 2016) (Figure 2). However, the role of cIAPs in polyubiquitination of RIPK2 upon exposure to NOD1 and NOD2 ligands is controversial, because other studies failed to reproduce the ubiquitination of RIPK2 by cIAPs (Damgaard et al., 2013; Stafford et al., 2018). In addition to K63-linked polyubiquitination, XIAP recruits the linear ubiquitin chain assembly complex (LUBAC) to induce NF-κB activation (Damgaard et al., 2012). RIPK2 is subjected to N-terminal methionine (Met1)-linked polyubiquitination by LUBAC after the interaction between RIPK2 and XIAP (Damgaard et al., 2012; Fiil et al., 2013). Thus, K63-linked polyubiquitination and recruitment of LUBAC are indispensable steps for NF-κB activation by RIPK2.

Various negative regulators of RIPK2 inhibit its polyubiquitination. For example, IRF4 and ATG16L1 physically interact with RIPK2 inhibiting its K63-linked polyubiquitination, thereby reducing pro-inflammatory cytokine production (Sorbara et al., 2013; Watanabe et al., 2014; Honjo et al., 2021). These studies support the concept that pro-inflammatory cytokine responses mediated by RIPK2 depend upon polyubiquitination and subsequent NF-κB activation.

## Autophagy and Receptor-Interacting Serine/Threonine Kinase 2

Autophagy is a process of regulated degradation of cytoplasmic constituents and intracellular pathogens (Mizushima et al., 2008; Virgin and Levine, 2009). It has been linked to mucosal host defense against invading pathogens in the gastrointestinal tract: mice deficient in ATG16L1, a critical regulator of the autophagic machinery, displayed impaired gut pathogen clearance (Kuballa et al., 2008; Travassos et al., 2010). Sensing of MDP or gut bacteria by intracellular NOD2 efficiently induces autophagy (Cooney et al., 2010; Travassos et al., 2010). Cooney et al. showed that the intact NOD2–RIPK2 pathway mediates autophagy in an ATG16L1-dependent manner in human DCs. However, Travassos et al. provided evidence that ATG16L1-mediated autophagy does not require the activation of RIPK2 in mouse embryonic fibroblasts (MEFs) (Travassos et al., 2010). This discrepancy can be partially explained by the type of cell studied, i.e., DCs vs. MEFs. Thus, RIPK2 is also involved in mucosal host defense mechanisms through the induction of ATG16L1-dependent autophagy in human DCs.

## NOD2–RIPK2 and TLRs–RIPK2 Axes in Inflammatory Bowel Disease

Given that loss-of-function mutations in *NOD2* or *ATG16L1* are associated with the development of CD (Cho, 2008; Chen et al., 2009; Philpott et al., 2014; Caruso et al., 2020), it is plausible that signaling pathways mediated by intact NOD2 and ATG16L1 prevent chronic inflammation of the gastrointestinal tract. In fact, MDP activation of NOD2 reduced pro-inflammatory cytokine responses, such as the release of IL-6, TNF-α, and IL-12/23p40, by colonic lamina propria APCs upon subsequent exposure to TLR and NOD1/NOD2 ligands, thereby inhibiting the development of colitis induced by dextran sulfate sodium (DSS) and trinitrobenzene sulfonic acid (TNBS) (Watanabe et al., 2008; Watanabe et al., 2014; Udden et al., 2017). The inhibition of DSS-induced colitis by NOD2 activation required a molecular interaction between NOD2 and RIPK2, because administration of MDP failed to prevent colonic inflammation in mice deficient in RIPK2 (Bertrand et al., 2009). Moreover, mice deficient in NOD2 or RIPK2 displayed enhanced susceptibility to DSS-induced colitis due to intestinal dysbiosis (Couturier-Maillard et al., 2013). Thus, MDP activation of NOD2 and RIPK2 plays a protective role in the development of experimental IBD.

Regarding the molecular mechanisms accounting for the inhibition of colitogenic cytokine responses by the intact NOD2–RIPK2 pathway, we and others have identified IRF4 to be a critical negative regulator (Watanabe et al., 2008; Watanabe et al., 2014; Udden et al., 2017). MDP activation of NOD2 induced IRF4 expression in DCs through the interaction of NOD2 with RIPK2, and then IRF4 suppressed the production of pro-inflammatory cytokines by DCs upon subsequent exposure to TLR ligands (Watanabe et al., 2008; Watanabe et al., 2014; Cavallari et al., 2017; Udden et al., 2017). IRF4 induced by the MDP-mediated activation of NOD2 inhibited activation of NF-κB through a physical interaction with myeloid differentiation factor 88, TRAF6, and RIPK2, which are all key signaling molecules in TLR pathways (Watanabe et al., 2008; Strober et al., 2014; Udden et al., 2017) (Figure 2). Likewise, K63-linked polyubiquitination of RIPK2 and TRAF6 was efficiently prevented by IRF4 induced by the MDP-mediated activation of NOD2 (Watanabe et al., 2008; Watanabe et al., 2014) (Figure 2). IRF4 binds to the KD and INTD of RIPK2 and inhibits polyubiquitination (Figure 1). Considering that MDP and TLR ligands are derived from intestinal bacteria, the intact NOD2–RIPK2 pathway activated by gut microbiota likely maintains intestinal homeostasis through the negative regulation of pro-inflammatory cytokine responses mediated by TLRs. CD-associated *NOD2* mutations decrease the ability of NOD2 to induce IRF4 expression due to defective recognition of MDP. In turn, the attenuated IRF4 expression leads to the excessive pro-inflammatory cytokine responses mediated by TLRs, which cause chronic colonic inflammation (Strober et al., 2006; Strober et al., 2007; Strober and Watanabe, 2011). This idea is fully supported by our data obtained in a unique model of experimental IBD induced by colonization with *Escherichia coli* and subsequent adoptive transfer of CD4^+^ T cells specific for *E. coli* (Watanabe et al., 2006). NOD2-deficient mice were susceptible to this unique type of colitis due to the excessive production of pro-inflammatory cytokines via TLR2 activation, because the mice deficient in both NOD2 and TLR2 were protected from colitis (Watanabe et al., 2006). Therefore, these studies suggest that the intact NOD2–RIPK2 pathway activated by the gut microbiota protects mice from experimental IBD through the IRF4-mediated suppression of pro-inflammatory TLR pathways. Protection by the intact NOD2–RIPK2 pathway against experimental IBD required polyubiquitination of RIPK2 as MDP activation of NOD2 did not ameliorate DSS-induced colitis in mice deficient in cIAP2 or Pollino3, the E3 ligases for K63-linked polyubiquitination of RIPK2 (Bertrand et al., 2009; Yang et al., 2013).

MDP activation of NOD2 acts synergistically with TLR ligands to induce pro-inflammatory cytokine responses (Fritz et al., 2005; Kim et al., 2008; Pashenkov et al., 2019). APCs produce a large amount of pro-inflammatory cytokines upon concomitant stimulation with MDP and TLR ligands. This is different from the prior exposure to MDP, because simultaneous exposures to MDP and TLR ligands lead to the enhanced generation of pro-inflammatory cytokine responses (Fritz et al., 2005; Kim et al., 2008; Pashenkov et al., 2019). Such synergistic effects of NOD2 on TLRs-mediated pro-inflammatory responses may play important roles in host defense against microbial infections (Fritz et al., 2005; Kim et al., 2008; Pashenkov et al., 2019). In addition, lack of these synergistic effects in the presence of CD-associated NOD2 mutations may lead to the development of chronic inflammation, because of gut bacterial overgrowth.

NOD2 is constitutively expressed in Paneth cells, which are specialized epithelial cells located in the crypts of the ileal mucosa (Inohara et al., 2005; Chen et al., 2009). Recognition of MDP by NOD2 induces robust production of α-defensin, a prototypical anti-microbial peptide, through RIPK2 activation followed by the nuclear translocation of NF-κB subunits in Paneth cells (Inohara et al., 2005; Chen et al., 2009). Indeed, NOD2-deficient mice displayed impaired protection against oral infection with *Listeria monocytogenes* (Kobayashi et al., 2005). Consistent with these data, ileal mucosa of CD patients with *NOD2* mutations, have a reduced expression of α-defensin in Paneth cells (Wehkamp et al., 2004; Wehkamp et al., 2005). In addition, mice deficient in NOD2 or RIPK2 develop granulomatous inflammation in the ileum when inoculated with *Helicobacter hepaticus*, and the effects are accompanied by a diminished expression of α-defensin (Biswas et al., 2010). These data suggest that the intact NOD2–RIPK2 pathway functions as a critical regulator of mucosal host defense mechanisms against gut microbiota through the production of α-defensin, and that impaired activation of RIPK2 in the presence of CD-associated *NOD2* mutations predisposes hosts to chronic inflammation due to bacterial overgrowth (Inohara et al., 2005; Kobayashi et al., 2005; Chen et al., 2009; Philpott et al., 2014). However, others reported conflicting results. Ileal mucosa from NOD2-intact and NOD2-defcient mice express comparable levels of α-defensin (Shanahan et al., 2014). In addition, reduced expression of ileal α-defensin is not associated with the *NOD2* mutations in patients with CD (Simms et al., 2008).

As mentioned above, RIPK2 activation by NOD2 and TLRs plays protective and pathogenic roles in the development of experimental IBD, respectively. Therefore, it is logical to ask whether RIPK2 could be a therapeutic target in IBD. In this regard, we recently provided evidence that NOD1-and/or NOD2-independent activation of RIPK2 caused experimental IBD through TLRs-induced pro-inflammatory cytokine responses (Watanabe et al., 2019). The blockade of RIPK2 activation by an intrarectal administration of a plasmid harboring RIPK2-specific siRNA encapsulated in the hemagglutinating virus of Japan envelope efficiently prevented the development of DSS- and TNBS-induced colitis in mice with intact NOD1 and NOD2, whereas colitis was manifested in animals that received intrarectal administration of the plasmid carrying control siRNA (Watanabe et al., 2019). Intrarectal administration of the plasmid encoding RIPK2-specific siRNA markedly reduced the production of the pro-inflammatory cytokines IL-6, TNF-α, and IL-12/23p40 by colonic lamina propria APCs upon the subsequent exposure to TLR ligands *in vitro* (Watanabe et al., 2019). Interestingly, siRNA-mediated knockdown of RIPK2 expression inhibited the development of both DSS- and TNBS-induced colitis in a NOD1/NOD2-independent manner because mice deficient in both NOD1 and NOD2 or in NOD2 only displayed significantly attenuated levels of DSS- and TNBS-induced colitis following the intrarectal administration of the plasmid carrying RIPK2-specific siRNA (Watanabe et al., 2019). Thus, TLRs-dependent and NOD2-independent activation of RIPK2 mediated the development of experimental IBD. Consistent with this idea, Hollenbach et al. showed that inhibition of RIPK2 by intraperitoneal administration of SB203580 improved clinical and histological scores in DSS- and TNBS-induced colitis, which was accompanied by the downregulation of pro-inflammatory cytokine responses and NF-κB activation (Hollenbach et al., 2004; Hollenbach et al., 2005). Another study tested gefitinib as a RIPK2 inhibitor and found that spontaneous development of ileitis seen in SAMP1/YitFc mice was ameliorated by oral administration of the drug (Tigno-Aranjuez et al., 2014). WEHI-345, which was identified as a RIPK2 inhibitor by screening a proprietary library of 120 kinase inhibitors, delayed NF-κB activation by inhibiting polyubiquitination of RIPK2 and ameliorated experimental autoimmune encephalomyelitis (Nachbur et al., 2015). Salla et al. (2018) identified and characterized two novel RIPK2 inhibitors by using molecular modeling and chemoinformatics analysis (Salla et al., 2018). The degree of suppression in DSS-induced colitis was significantly greater in mice treated with these RIPK2 inhibitors than in those treated with gefitinib (Salla et al., 2018). Although these studies utilizing RIPK2 inhibitors have not clearly shown whether such small molecules suppressed the activity of the NOD2–RIPK2 and TLRs–RIPK2 pathways to improve inflammation, their therapeutic and preventive effects seen in experimental IBD allow considering RIPK2 as a new therapeutic target in human IBD.

The clinical relevance of RIPK2 activation was examined by using colonic biopsy samples obtained from CD and UC patients during colonoscopy (Watanabe et al., 2019). Expression of RIPK2 as determined by qPCR was much higher in colonic lesions of patients with CD and UC than in normal colonic mucosa (Watanabe et al., 2019). Expression levels of RIPK2-related signaling molecules such as cIAP1, cIAP2, TRAF6, and TAK1 were significantly higher in patients with CD and UC than those in normal colonic mucosa (Watanabe et al., 2019). Although RIPK2 expression positively correlated with that of pro-inflammatory cytokines in the colonic mucosa of patients with CD and UC, enhanced expression of NOD1 and NOD2 was not observed in IBD patients (Watanabe et al., 2019). Consistent with qPCR data, immunofluorescence studies showed that colonic DCs expressing RIPK2 and cIAP2 produce TNF-α. Moreover, the intensity of the molecular interaction between cIAP2 and RIPK2 and between RIPK2 and TAK1 is positively correlated with expression levels of IL-6 and TNF-α in the inflamed colonic mucosa of patients with CD and UC (Watanabe et al., 2019). Thus, our study highlighted the importance of RIPK2 activation in the generation of colitogenic cytokine responses in human IBD. Excessive activation of RIPK2 has also been observed in the inflamed mucosa of pediatric CD patients (Negroni et al., 2009). In line with these data, GSK583, a highly potent and selective inhibitor of RIPK2, efficiently suppressed the spontaneous release of pro-inflammatory cytokines in colonic biopsy samples obtained from CD patients (Haile et al., 2016.). Taken together, these studies utilizing human IBD samples fully support the view that activation of RIPK2 plays a pathogenic role in the development of both murine and human IBD by promoting the production of pro-inflammatory cytokines. Therefore, RIPK2 can be a novel therapeutic target in human IBD.

## ATG16L1–RIPK2 Axis in Inflammatory Bowel Disease

APCs or epithelial cells expressing CD-associated *ATG16L1* mutations displayed diminished autophagic responses to invading the gut microbiota (Kuballa et al., 2008; Cooney et al., 2010; Travassos et al., 2010). NOD2 activation induced by MDP or intestinal bacteria is a potent trigger of autophagy (Cooney et al., 2010; Travassos et al., 2010). DCs isolated from CD patients bearing CD-associated *NOD2* mutations showed defective autophagy upon the exposure to MDP or *Salmonella enterica* (Cooney et al., 2010). Thus, impaired autophagy in response to invasion of the gut microbiota, underlies the immunopathogenesis of CD in the presence of *NOD2* or *ATG16L1* mutations (Cho, 2008; Kaser and Blumberg, 2011; Salem et al., 2015).

Diminished autophagy caused by CD-associated *ATG16L1* mutations or a complete lack of the *ATG16L1* gene results in enhanced pro-inflammatory cytokine responses, including excessive release of IL-1β, IL-6, and TNF-α, triggered by TLR2 and TLR4 ligands (Saitoh et al., 2008; Lassen et al., 2014; Murthy et al., 2014). Thus, enhanced pro-inflammatory cytokine responses to TLR ligands caused by defective autophagy drive chronic inflammation in CD patients with *ATG16L1* mutations. In other words, intact ATG16L1 pathways maintain intestinal homeostasis through the negative regulation of TLRs-mediated pro-inflammatory responses. However, the molecular mechanisms by which such cytokine responses are negatively regulated by intact ATG16L1 pathways are poorly understood.

Given that RIPK2 functions as a signaling molecule downstream of not only NOD1 and NOD2 but also of TLRs, it is possible that ATG16L1 negatively regulates pro-inflammatory cytokine responses mediated by the TLRs–RIPK2 pathway. We have demonstrated previously that ATG16L1 binds to the KD of RIPK2 (Honjo et al., 2021) (Figure 1). The TLR2-mediated NF-κB activation was significantly suppressed by the overexpression of intact *ATG16L1*, but not by the overexpression of *ATG16L1* harboring CD-associated mutations, in reporter gene assays. In human monocyte–derived DCs, MDP activation of NOD2 induced physical interaction between ATG16L1 and RIPK2 and negatively regulated TLR2-mediated NF-κB activation and pro-inflammatory cytokine responses by inhibiting the interaction between TLRs and RIPK2 (Honjo et al., 2021). Consistent with the data from a previous report (Sorbara et al., 2013), ATG16L1 binding to RIPK2 suppressed NF-κB activation by downregulating polyubiquitination of RIPK2 (Honjo et al., 2021) (Figure 2). Importantly, ATG16L1 acts synergistically with IRF4 to inhibit RIPK2 polyubiquitination. These data suggest that ATG16L1 functions as a negative regulator of the TLR2–RIPK2 pathway.

The clinical relevance of these findings was examined in samples obtained from patients with CD and UC. The percentages of colonic DCs expressing ATG16L1 were inversely correlated with the expression levels of IL-6 in the colon of both CD and UC patients and with TNF-α expression level in the colon of CD patients only (Honjo et al., 2021). Induction of remission was associated with the accumulation of colonic DCs expressing ATG16L1 in CD patients (Honjo et al., 2021), which was consistent with aggregated DSS-induced colitis in mice with DC-specific *Atg16l1* deletion (Zhang et al., 2017; Honjo et al., 2021). Together, these data suggest that the interaction between ATG16L1 and RIPK2 induced by NOD2 activation prevents colitogenic cytokine responses mediated by TLRs. It should be noted, however, that confirmation of this idea awaits future studies that should utilize samples from CD patients with *ATG16L1* mutations. Plantinga et al. (2011) showed comparable levels of pro-inflammatory cytokine production by peripheral blood mononuclear cells isolated from CD patients with and without *ATG16L1* mutations upon the stimulation with TLR2 and TLR4 ligands (Plantinga et al., 2011).

Epithelial cells express functional NOD2, RIPK2, and ATG16L1. MDP activation of NOD2 induces autophagy and mediates the anti-bacterial response (Homer et al., 2010). Epithelial cells expressing CD-associated *NOD2* or *ATG16L1* mutations, exhibit impaired autophagy when exposed to MDP (Homer et al., 2010). Induction of autophagy mediated by NOD2 and ATG16L1 requires intact RIPK2, because epithelial cells with defective expression of RIPK2 also exhibit a reduced induction of autophagy (Homer et al., 2010). Collectively, the NOD2-RIPK2-ATG16L1 axis operating in epithelial cells contributes to the maintenance of intestinal homeostasis through induction of autophagy.

## Recent Progress in Receptor-Interacting Serine/Threonine Kinase 2 Inhibitors

Several RIPK2 inhibitors have been used for the treatment of experimental colitis. These RIPK2 inhibitors are believed to inhibit kinase activity of RIPK2 (Hollenbach et al., 2005; Tigno-Aranjuez et al., 2014; Nachbur et al., 2015; Salla et al., 2018). However, recent studies provide evidence that kinase activity of RIPK2 is dispensable for NOD2 signaling, and that RIPK2 inhibitors inhibit the polyubiquitination of RIPK2 through disrupting the interaction between RIPK2 and XIAP (Goncharov et al., 2018; Hrdinka et al., 2018; Suebsuwong et al., 2020). Therefore, it is likely that RIPK2 inhibitors prevent experimental colitis through the suppression of polyubiquitination of RIPK2.

Mice deficient in NOD2 or RIPK2 are more susceptible to DSS or TNBS-induced colitis; however, the administration of RIPK2 inhibitors ameliorates chronic colonic inflammation. Activation of RIPK2 plays dual roles in the development of experimental colitis. We speculate that the NOD2-RIPK2 and TLRs-RIPK2 pathways play anti-inflammatory and pro-inflammatory roles, respectively, in the development of experimental colitis. This idea is fully supported by our recent report in which NOD1 or NOD2-independent activation of RIPK2 mediates experimental colitis (Watanabe et al., 2019). Expression of RIPK2 and its signaling molecules, such as cIAP2, TRAF6, and TAK1, was markedly elevated in the colonic mucosa of patients with UC or CD compared to that in the healthy colonic mucosa, while the expression of NOD2 decreased (Watanabe et al., 2019). These studies strongly suggest that the interaction between TLRs and RIPK2, rather than that between NOD2 and RIPK2, could be a novel therapeutic target for IBDs. However, the possibility of patients with IBDs being successfully treated with RIPK2 inhibitors awaits the development of RIPK2 inhibitors that inhibit the interaction between TLRs and RIPK2.

## Concluding Remarks

The loss-of-function mutations in *NOD2* and *ATG16L1* are associated with development of CD. Considering that signaling pathways mediated by NOD2 and ATG16L1 merge at the level of RIPK2 in APCs, it is likely that intact NOD2–ATG16L1–RIPK2 pathways maintain intestinal immune homeostasis. Recent studies have provided evidence that the intact NOD2–RIPK2 pathway downregulates colitogenic pro-inflammatory cytokine responses mediated by the TLRs–RIPK2 pathways through the activation of IRF4 and ATG16L1. Mechanistically, IRF4 and ATG16L1 downregulate TLRs-induced pro-inflammatory cytokine responses by inhibiting polyubiquitination of RIPK2. Enhanced activation of RIPK2 was verified in the inflamed colonic mucosa of patients with CD and UC. Small molecules inhibiting RIPK2 have been identified, and their administration successfully treated experimental IBD. These findings strongly suggest that patients with UC and CD might be treated with RIPK2 inhibitors.
